# Changes in Muscle Mass and Bone Density and Their Relationship After Total Knee Arthroplasty

**DOI:** 10.3390/jcm13226700

**Published:** 2024-11-07

**Authors:** Juneyoung Heo, Han-Seung Koh, Chang Hyun Nam, Dong-Chan Lee, Ji-Hoon Baek, Hye Sun Ahn, Su Chan Lee

**Affiliations:** 1 Joint & Arthritis Research, Department of Neurosurgery, Himchan Hospital, Seoul 07999, Republic of Korea; juneyoungheo@gmail.com (J.H.); nskhs1@naver.com (H.-S.K.); akula02@naver.com (D.-C.L.); 2 Joint & Arthritis Research, Department of Orthopaedic Surgery, Himchan Hospital, Seoul 07999, Republic of Korea; changcape@naver.com (C.H.N.); jihoon011@naver.com (J.-H.B.); ahs0614@naver.com (H.S.A.)

**Keywords:** knee, osteoarthritis, arthroplasty, sarcopenia, osteoporosis, bone mineral density

## Abstract

**Purpose:** To investigate the effects of total knee arthroplasty (TKA) on muscle mass and bone density in end-stage knee osteoarthritis (OA). **Methods:** This prospective study was conducted on 111 patients with Kellgren–Lawrence grade 4 knee OA who underwent TKA after failing to respond to conservative treatment for more than 3 months at a single institution from June 2022 to May 2023. Appendicular lean mass index (ALMI) and bone mineral density (BMD) were measured using dual-energy X-ray absorptiometry before and every 6 months after surgery. The average follow-up period was 15.5 ± 2.31 months (range, 11.6–24 months). **Results:** During the follow-up period after TKA, the ALMI increased relatively continuously and consistently. The BMD of the L-spine and proximal femur did not change significantly until 12 months after TKA surgery but began to increase steeply after 12 months and slowed down after 18 months. The increase in muscle mass showed a significant positive correlation with the increase in BMD. **Conclusions:** Muscle mass gradually increased after TKA for end-stage knee OA, whereas bone density also increased but not until 12 months after surgery. The significant positive correlation between the increase in muscle mass and bone density suggests that the increase in muscle mass after TKA may be one of the causes of the increase in bone density.

## 1. Introduction

Osteoporosis and sarcopenia, which are primarily observed in older people, are systemic musculoskeletal disorders characterized by loss of bone and muscle masses. The general criterion recommended by the World Health Organization for older people is age ≥65 years. Osteoarthritis (OA) is also the most common chronic joint disease among older people. Considering that the components of the musculoskeletal system, such as bones, muscles, and cartilage, are closely connected to each other, they closely influence each other. Obesity, age, sex, and history of joint injuries are well-known risk factors for knee OA, and bone density and muscle weakness also play crucial roles [1].

As we age, our bodies undergo changes leading to bone and muscle loss. 11β-hydroxysteroid dehydrogenase, which converts inactive cortisone into cortisol, increases with aging [2]. The higher the activity of 11β-hydroxysteroid dehydrogenase, the lower the muscle strength [3]. With age, the amounts of dehydroepiandrosterone (DHEA) and dehydroepiandrosterone sulfate (DHEAS) secreted by the adrenal gland gradually decrease, and they are converted into androgen and estrogen in peripheral tissues [4,5]. DHEA and DHEAS are associated with improved physical functions—such as muscle strength and bone density—as well as anti-inflammatory and immune-regulatory functions [6,7]. In particular, the imbalance between bone formation and resorption due to estrogen deficiency causes connectivity loss in trabecular bone and bone cortex thinning [8]. In addition, vitamin D levels decrease with age, which may lead to decreased intestinal calcium absorption and secondary hyperparathyroidism [9]. Circulatory parathyroid hormone potentially increases with age, independent of vitamin D, calcium, phosphate, and kinase functions. The resulting secondary hyperparathyroidism is common in postmenopausal women, leading to decreased bone density [10]. Additionally, decreases in growth hormone and insulin-like growth factor during aging are associated with bone loss [11,12].

The pro-inflammatory environment caused by decreased physical function and physical activity can decrease muscle size, increase cartilage destruction, and ultimately negatively affect bones [13]. Decreased physical activity increases the concentration of adipose cells between muscle fibers and the production of adipokines such as leptin, causing inflammation. This condition can lead to OA through joint and cartilage destruction [14].

The knee OA and sarcopenia interaction is multifactorial. The interactions among physiological factors such as proinflammatory cytokines and hormones originating from chronic low-grade inflammation and biomechanical effects originating from lifestyle are complex. Among them, irisin, the Pl3K/AKT pathway, and caspase-3 are potential factors causing sarcopenia to induce knee OA. Irisin levels are significantly reduced in patients with sarcopenia regardless of BMI and age and are considered an independent predictor of sarcopenia [15,16]. Irisin can delay cartilage damage in the damaged cartilage. When irisin levels are reduced in sarcopenia, articular cartilage damage occurs more easily [1]. Using this mechanism, irisin reduction due to sarcopenia can accelerate knee OA progression. When caspase-3 protein is activated in the Pl3K/AKT pathway, which is essential for normal metabolism of the joint tissue, protein synthesis decreases and protein degradation in the muscles increases, causing muscle atrophy [17,18]. Muscle atrophy induced by caspase-3 can also promote insulin resistance, aggravated by the ability to decompose inflammatory factors in the joint, which leads to synovial inflammation and OA. Insulin resistance can ultimately cause OA by inducing apoptosis in synovial cells through the TLR-4 pathway [19].

Although several studies have investigated the effects of osteoporosis and sarcopenia on knee OA, only one study has examined the effects of total knee arthroplasty (TKA) on muscle mass after, whereas three studies have investigated its effects on osteoporosis. Despite the close relationship between muscles and bones, no study to date has concurrently analyzed the changes in muscle mass and bone density after TKA.

The current study was therefore conducted to determine the effects of TKA for end-stage knee OA on muscle mass and bone density. Accordingly, we hypothesized that knee OA would be an important risk factor for osteoporosis and sarcopenia.

## 2. Materials and Methods

This prospective study was approved by the Institutional Review Board of Himchan Hospital, and the personal information of all participants was protected. This study was conducted on patients who visited a single institute from June 2022 to May 2023. All patients who visited the hospital underwent history taking, physical examination, and bilateral knee roentgenography to diagnose knee osteoarthritis. Patients were not asked what sports they played as hobbies before surgery; no patient was a current or former athlete.

Exclusion criteria were (1) patients using steroids for a long period of time due to illness, etc.; (2) those currently being treated for osteoporosis; (3) those with any neurologic issues (significant cognitive impairment, paralysis, etc.) or other illnesses (heart failure, severe lung disease, etc.) that limit exercise and daily active activity even after surgery; (4) history of connective tissue disorders or myositis; (5) history of knee arthroplasty; and (6) history of alcoholism or drug abuse.

Among patients with Kellgren–Lawrence (K–L) grade 4 knee OA who failed to show improvement in their symptoms despite conservative treatment, including nonsteroidal anti-inflammatory drugs (NSAIDs) and physical therapy for at least 3 months, those who agreed to undergo TKA were included in this study. We recommended moderate-to-high-intensity aerobic exercises that the patient could tolerate two times a week. Patients were also informed about exercises that could strengthen the quadriceps muscle and encouraged to perform them at least three times a week, including microwave diathermy, ultrasound therapy, transcutaneous electrical nerve stimulation, and range-of-motion exercises for reducing pain and improving function. All patients underwent preoperative measurement of appendicular lean mass index (ALMI) and bone mineral density (BMD) using dual-energy X-ray absorptiometry (DEXA). Several methods can be explored to evaluate muscle mass and BMD; however, herein, AMLI and BMD, measured using DEXA, were used, which is a typical method. Each way of evaluating muscle mass has limitations. The most effective method to date is measuring lean mass using DEXA, and because the European Working Group on Sarcopenia in Older People and the Asian Working Group for Sarcopenia (AWGS) use it to measure muscle mass, ALMI using DEXA was employed herein. BMD measured via DEXA has limitations, such as bone density measurements being higher in cases of obesity or posture during bone density measurements affecting the results. However, measuring BMD by DEXA is the most widely used method worldwide and is accepted as a standardized method for measuring bone density. BMD was measured using the T-score of the spine and femur according to the suggestion of the International Society for Clinical Densitometry [20]. For the spine, the average value of more than two segments of the lumbar spine was measured, whereas for the hip joint, the proximal femur and femoral neck were measured. A diagnosis of osteoporosis was established when the lowest T-score of the spine and hip joint was 2.5 or lower. Muscle mass was measured using DEXA according to the criteria of the AWGS, with sarcopenia being defined as low muscle mass (i.e., the ALMI of <7.0 and <5.4 kg/m^2^ in men and women, respectively) [21]. The AWGS defines sarcopenia as low muscle mass, low muscle strength, and low physical performance. Possible effects on muscle strength should be considered while evaluating sarcopenia [22,23,24,25,26]. Herein, patients with K-L grade 4 knee OA who did not respond to conservative therapy could not walk properly because of pain. These patients walked with a limp because of knee pain or had to rest because of pain after walking a short distance; therefore, their gait speed was significantly reduced compared with their muscle mass. In addition, these patients had increased handgrip strength as they walked around with a cane. Because of these characteristics observed in the patient group, gait speed and handgrip strength, which could affect muscle strength, were not measured. In this study, sarcopenia was defined based solely on the ALMI, which indicates muscle mass.

After TKA, NSAIDs were administered for pain control until 2 to 3 months after surgery, and patients performed weight-bearing exercises within the tolerable range immediately after surgery. Steroids or antiosteoporotic drugs can affect the results of muscle mass and bone density tests. Even if the same steroids or antiosteoporotic drugs are given to patients with similar physical conditions, changes in these patients are unpredictable. Accordingly, these drugs are judged to exert a significant effect on the research results; therefore, they were not used during the study period. Afterward, the ALMI and BMD were measured using DEXA every 6 months.

Variables were statistically analyzed using the paired t-test, analysis of variance, and Pearson correlation coefficient. The Statistical Package for the Social Sciences version 27 (SPSS version 27, Chicago, IL, USA) was used for all statistical analyses. All p values were two-sided, with a value of <0.05 indicating statistical significance.

## 3. Results

A total of 254 patients with K-L grade 4 knee OA visited our hospital, among whom 208 underwent TKA due to failure to respond to conservative treatment for more than 3 months. After excluding 97 patients who satisfied the exclusion criteria, 111 patients were ultimately selected for the prospective study (Table 1). The patients had a mean age of 73.2 ± 4.5 years (range, 61–86 years) and were predominantly female (81%). Bilateral TKA was performed in 58 patients, whereas unilateral TKA was performed in 53 patients. The mean follow-up period was 15.5 ± 2.31 months (range, 11.6–24 months) (Table 1). No patient who underwent surgery developed serious complications. One patient had a postoperative hematoma that did not improve after conservative treatment for a week but improved without complications after surgical treatment. Three patients showed dehiscence in a part of the operative wound, which improved after repair. A total of 12 patients complained of sensory loss in the lateral knee; however, the condition was tolerable. Two patients complained of stiffness in the operated knee; by performing continuous passive motion every day, the symptoms improved within 1 month. Five patients complained of intermittent crepitus in the operated knee but did not experience any discomfort.

The prevalence of sarcopenia among our participants was 58.5% (65 patients), whereas the prevalence of osteoporosis was 51.3% (57 patients). The prevalence of sarcopenia (women 60% vs. men 53%) and osteoporosis (women 65% vs. men 13%) was higher in women than in men. The prevalence of sarcopenia among all female patients was 60% (49 patients), which was higher than that in men (53%, 16 patients). Meanwhile, the prevalence of osteoporosis among all female patients was 65% (53 patients), which was higher than that in men (13%, 5 patients).

The mean preoperative ALMI was 5.37 ± 0.75, which was higher in men than in women. The mean BMD of the L-spine was −1.61 ± 1.2, whereas the mean BMD of the proximal femur was −2.10 ± 0.89, with both BMD values being higher in men than in women. The mean body mass index (BMI) was 26.55 ± 3.47, with no significant difference between men and women.

In DEXA performed 6 months after TKA surgery, ALMI increased by 0.133 ± 0.090; however, the increases in L-spine BMD and proximal femur BMD were 0.05 ± 0.074 and −0.23 ± 0.13, respectively, showing little change or a slight decrease (Table 2). At 12 months after surgery, ALMI further increased to 0.313 ± 0.22, and L-spine BMD and proximal femur BMD increased by 0.53 ± 0.12 and 0.11 ± 0.15 compared with that before surgery, showing a gradual increase. The increase in ALMI continued to increase at 18 and 24 months after surgery, reaching 0.382 ± 0.18 and 0.401 ± 0.16, respectively. During the same period, L-spine and proximal femur BMD increased even more steeply, reaching 0.250 ± 0.10 and 0.133 ± 0.12 at 24 months after surgery, respectively.

Compared with patients who underwent unilateral TKA, those who underwent bilateral TKA had lower muscle mass and bone density; however, the difference was not significant. Additionally, no difference was found in the amount of increase after TKA surgery (Table 3). The preoperative mean ALMI of patients with bilateral OA was 5.26 ± 0.87, which was lower than the ALMI value of 5.48 ± 0.67 of patients who underwent unilateral TKA. However, the difference was not significant. The preoperative L-spine and proximal femur of patients who underwent bilateral TKA were −1.53 ± 1.1 and −2.01 ± 0.95, respectively, which were higher than −1.69 ± 1.32 and −2.17 ± 0.81 of patients who underwent unilateral TKA; however, the difference was not significant. No difference was noted in the amount of increase in ALMI, L-spine BMD, and proximal femur BMD between the two groups. No difference was observed between the two groups when adjusted for sex, age, and follow-up period after surgery.

The Pearson correlation coefficient between changes in muscle mass and L-spine BMD was 0.106, with a two-sided significance probability of 0.05. The 95% confidence interval was −0.067 to 0.274. The Pearson correlation coefficient between changes in muscle mass and proximal femur BMD was 0.12, with a two-sided significance probability of 0.03. The 95% confidence interval was −0.16 to 0.184.

## 4. Discussion

**Figure 1:**
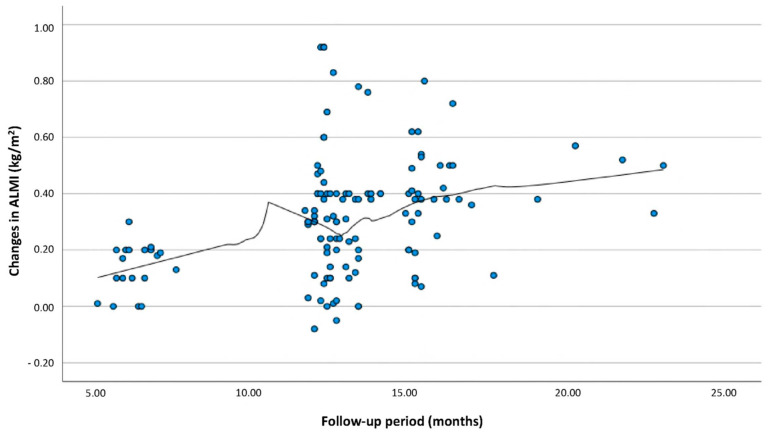
Changes in appendicular lean mass index during the follow-up period after TKA.

The current study found that the ALMI increased continuously during the follow-up period after TKA. The rate at which the ALMI increased remained relatively constant throughout the follow-up period (Figure 1). One study conducted in Hong Kong revealed that the ALMI increased by approximately 0.2 kg/m^2^ 12 months after TKA [27]. Moreover, despite the slight difference compared to the results observed in this study, both studies share a common feature, which is that the ALMI continuously increased starting immediately after surgery.

**Figure 2:**
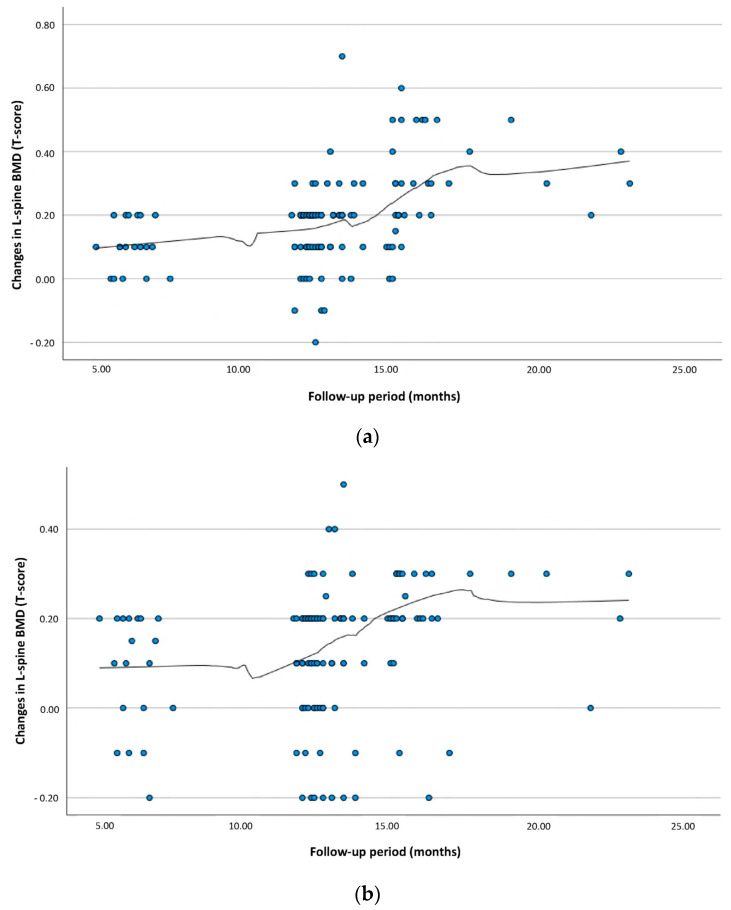
Changes in bone mineral density (BMD) during the follow-up period after TKA; (**a**) L-spine BMD, (**b**) proximal femur neck BMD.

Our results showed that the BMD of the L-spine and proximal femur did not change significantly until 12 months after TKA surgery but began to increase steeply after 12 months and subsequently slowed down after 18 months (Figure 2 and Table 2). This result is quite consistent with those reported in previous studies [28,29,30], which observed changes in BMD after TKA. Meanwhile, studies by Kim et al. and Yasar et al. found no significant change in BMD after TKA, although both studies had a short follow-up period of 12 months [28,29]. The study by Ishii et al. in 2000 showed that the BMD of the proximal femur was 45–59% higher than the predicted age-related loss after an average follow-up of 48 months after TKA [30]. However, the study by Ishii et al. found that BMD initially decreased for several months after TKA but then subsequently increased, recovering to preoperative BMD levels 1–2 years after TKA. A comprehensive review of the results of our study and previous studies suggests that bone density may decrease or remain unchanged for up to 12 months after TKA and that bone density can be expected to increase after 12 months.

Significant positive correlations were observed between muscle mass and L-spine BMD and between muscle mass and proximal femur BMD (*p* value < 0.001), with correlation coefficients of 0.106 and 0.012, respectively. In other words, a greater increase in muscle mass was correlated with a greater increase in bone density. For 1 to 2 months after TKA, activity may decrease relative to that before surgery due to pain and swelling at the surgical site, decreased range of motion, etc. After that, however, evidence suggests that the amount of activity gradually increases, leading to an increase in muscle mass at the first follow-up observation period of 6 months. Considering that the increase in activity did not directly induce changes in bone density but did occur first after the increase in muscle mass and that the increase in muscle mass was positively correlated with the increase in bone density, our findings suggest that the increase in muscle mass after TKA surgery could be one of the causes for the increase in BMD.

Patients with and without sarcopenia before surgery showed an increase in muscle mass, with no difference in the extent of the increase. This finding is consistent with that reported in previous research [27]. The presence or absence of osteoporosis before surgery did not affect the amount of BMD increase after surgery. Sex, age, and unilateral or bilateral TKA did not affect the changes in muscle mass and BMD after surgery.

Pain due to knee OA leads to low physical activity, which is an important risk factor for sarcopenia [22]. Hence, knee OA can cause a decrease in muscle mass. A comprehensive review of recent studies showed that the prevalence of sarcopenia was higher among patients with knee OA than among the general population [31]. In particular, a comparison between Korean elderly female patients with severe knee OA awaiting TKA and the general population revealed that the prevalence of severe sarcopenia based on the AWGS criteria was approximately seven times higher in the former [32]. Conversely, some research results also show that sarcopenia predisposes patients to the development and progression of knee OA [33]. Thus, sarcopenia and knee OA have been considered to have a negative relationship with each other. Physical activity also affects bones, with evidence showing that physical activity preserves bone density [34] and reduces the risk of hip fracture while promoting an increase in muscle mass among middle-aged and older individuals [35]. Long-term immobilization has been identified as a risk factor for a decrease in muscle mass and bone density [36]. These research results suggest that physical activity plays a critical role in muscle mass and bone density.

The interaction between the muscles and bones can be explained by the “mechanostat” theory, which states that heightened loading on the bone triggers osteoblasts to create bone. Conversely, a prolonged period of disuse triggers osteoclasts to proceed with bone resorption. In such a context, muscles are an important factor in the application of mechanical force to the bone, which facilitates bone formation when this force exceeds a set limit [37]. This phenomenon has been attributed to the stretching of the periosteum and collagen fibers due to an increase in muscle mass, thereby stimulating bone growth [38]. In addition, a protein called irisin secreted from the muscles during exercise promotes osteocyte survival and inhibits osteoclast activity [39]. As such, muscle activity and the mechanical force that muscles exert on bone play a critical role in bone formation and maintenance.

Recently, researchers have coined the term “osteosarcopenia” to describe the co-occurrence of osteoporosis and sarcopenia [40]. Indeed, accumulating evidence has shown significant pathophysiologic overlap between osteoporosis and sarcopenia; thus, treatments targeting both conditions are being studied [41]. Given these recent research results, osteoporosis and sarcopenia should always be considered together.

Several studies have shown that the prevalence of sarcopenia is higher in patients with knee OA than in patients without OA [27,32,42], with our study showing similar results. Studies conducted in Asia have shown that the prevalence of sarcopenia in the general population was 6.7–18.6% in older men and 0.1–23.6% in older women [43,44,45,46]; however, the prevalence of knee OA in patients with knee OA was 32.8–35.5%. In the current study, the prevalence of sarcopenia in patients with end-stage knee OA was 58.5%, which was more than twice that in the general elderly population. Moreover, our results showed that women were particularly vulnerable to sarcopenia. Alternatively, a 2020 study showed that the prevalence of osteoporosis in Korea was 7.5% and 18.0% among men in their 60s and 70s and 36.6% and 68.5% among women in their 60s and 70s, respectively [47]. Notably, these figures did not significantly differ from those observed in the current study after adjusting for age and sex ratio.

This study is the first to observe changes in muscle mass and bone density after TKA in patients with end-stage knee OA. One limitation of this study was that handgrip strength and gait speed, which are measures of muscle strength and physical performance, were not measured when diagnosing sarcopenia. In addition, given that this study was conducted at a single institution, the generalizability of our results may be limited. This study showed the temporal causal relationship between changes in muscle mass and bone density after TKA, as well as its significance. Sex is a major factor affecting BMD and ALMI, and the inclusion of about twice as many women as men is a limitation of this study. Similarly, the age of patients, which significantly affects BMD and ALMI, varied, which could have affected the results. Additional clinical studies and laboratory and animal experiments on the relationship between muscle mass and bone density are warranted in the future.

## 5. Conclusions

Our findings showed that muscle mass gradually increased after TKA surgery for end-stage knee OA, whereas bone density also increased but not until after 12 months. In addition, the significant positive correlation between the increase in muscle mass and the increase in bone density suggests that the increase in muscle mass after TKA surgery may be one of the causes of the increase in bone density.

## Figures and Tables

**Table 1:** Demographic characteristics and preoperative muscle mass and osteoporosis data.

	Final Cohort
Sex (male–female)	30:81
Age (years) (mean ± SD)	73.19 ± 4.53 (61–86)
Male	72.93 ± 3.34
Female	73.30 ± 4.91
TKA (n)	
Unilateral	53
Bilateral	58
BMI (kg/m^2^)	26.55 ± 3.47
Male	26.36 ± 3.43
Female	26.61 ± 3.51
ALMI	5.37 ± 0.75
Male	6.76 ± 0.96
Female	5.26 ± 0.64
L-spine BMD	−1.61 ± 1.20
Male	−0.33 ± 1.02
Female	−2.08 ± 0.87
Proximal femur BMD	−2.10 ± 0.89
Male	−1.36 ± 1.05
Female	−2.37 ± 0.64
Preoperative sarcopenia (n)	65
Male	16
Female	49
Preoperative osteoporosis (n)	57
Male	4
Female	53

SD, standard deviation; TKA, total knee arthroplasty; BMI, body mass index; BMD, bone mineral density.

**Table 2:** Changes in muscle mass and bone mineral density during the follow-up period after total knee arthroplasty.

	Changes in ALMI	Changes in L-Spine BMD	Changes in Proximal Femur BMD
6 months	0.133 ± 0.090	0.005 ± 0.074	−0.023 ± 0.13
12 months	0.313 ± 0.22	0.053 ± 0.12	0.011 ± 0.15
18 months	0.382 ± 0.18	0.169 ± 0.16	0.095 ± 0.12
24 months	0.401 ± 0.16	0.250 ± 0.10	0.133 ± 0.12

ALMI, appendicular lean mass index; BMD, bone mineral density.

**Table 3:** Comparison of muscle mass, bone density, and changes in patients who underwent unilateral and bilateral TKAs.

	Unilateral TKA	Bilateral TKA	*p* Value
Preoperative ALMI	5.48 ± 0.67	5.26 ± 0.87	0.15
Changes in ALMI after TKA	0.32 ± 0.22	0.29 ± 0.20	0.36
Preoperative L-spine BMD	−1.69 ± 1.32	−1.53 ± 0.11	0.24
Changes in L-spine BMD after TKA	0.17 ± 0.14	0.18 ± 0.14	0.69
Preop proximal femur BMD	−2.17 ± 0.81	−2.01 ± 0.95	0.87
Changes in proximal femur BMD after TKA	0.12 ± 0.15	0.14 ± 0.14	0.55

ALMI, appendicular lean mass index; BMD, bone mineral density; TKA, total knee arthroplasty.

## Data Availability

The data presented in this study are available upon reasonable request from the corresponding author.
